# Influencing Factors of High-Risk Human Papillomavirus Infection and DNA Load According to the Severity of Cervical Lesions in Female Coal Mine Workers of China

**DOI:** 10.7150/jca.29034

**Published:** 2019-10-06

**Authors:** Yuanjing Lyu, Ling Ding, Tao Gao, Ying Li, Li Li, Ming Wang, Yang Han, Jintao Wang

**Affiliations:** 1 Department of Epidemiology, School of Public Health, Shanxi Medical University, Taiyuan, China.; 2 Department of Preventive Medicine, Robert H. Lurie Comprehensive Cancer Center, Feinberg School of Medicine, Northwestern University, Chicago, America.; 3 Department of pathology, Jincheng General Hospital, Jincheng, China.

**Keywords:** HR-HPV infection, influencing factors, HR-HPV DNA load, female coal mine workers

## Abstract

High-risk human papillomavirus (HR-HPV) plays an aetiological role in the progression of cervical cancer and precancerous lesions. Determining the risk factors of HR-HPV infection is useful for HR-HPV infection surveillance and control. We aimed to explore the influencing factors of HR-HPV infection in female coal mine workers, and to evaluate the associations between HR-HPV DNA load and cytological and histological changes of cervix. In total 6,325 participants completed standard questionnaire on potential influencing factors of HR-HPV infection and underwent gynecological examinations, HPV test as well as Thinprep cytology test (TCT). 1,512 women with positive results of HPV and/or TCT were referred to colposcopy with biopsy and histological examination. HR-HPV DNA load was evaluated by Digene second generation hybrid capture (HC2) assay. Multiple unconditional logistic regression analysis was used to determine the influencing factors for HR-HPV infection. Of 6,325 study participants, 1,405 (22.2%) were HR-HPV positive. HR-HPV infection rate was higher in women aged 30-50 years, with lower education level, working inside the mines and engaging in shift work. Risk factors for HR-HPV infection in female coal mine workers included contraception (OR=1.395, 95%CI=1.102-1.458), previous artificial abortion (OR=1.603, 95%CI=1.202-1.856), working inside the mines (OR=1.230, 95%CI=1.056-1.528) and history of gynecological diseases (OR=1.198, 95%CI=1.001-1.462), while menopause was a protective factor (OR=0.402, 95%CI=0.306-0.507). The HR-HPV DNA load significantly increased with the severity of cervical cytological (*χ*^2^_trend_=177.372, *p*<0.001) and histological (*χ*^2^_trend_=194.501, *p*<0.001) changes. The results indicated that HR-HPV infection is highly prevalent in female coal mine workers in China. Contraception, artificial abortion, working inside the mines and gynecological diseases could increase the risk of HR-HPV infection in these women. HR-HPV DNA load might predict risks of cervical precancerous lesions and cancer. Our findings could provide scientific basis for reducing the risk of HR-HPV infection and cervical cancer in this vulnerable population.

## Introduction

Cervical cancer is the fourth most common cancer among females worldwide 1. In China, there were 98,900 new cases and 30,500 deaths from cervical cancer in 2015, accounting for respectively 18.7% and 11.5% of all cervical cancer cases and deaths worldwide 2. The causal role of high-risk human papillomavirus (HR-HPV) in cervical carcinogenesis has been approved by various studies 3,4. HPVs, with an overall prevalence of approximately 10% (1.4-25.6%) worldwide, are categorized into HR-HPV and low-risk HPV (LR-HPV) 5. Geographical differences exist in the prevalence of HPV. According to a pooled analysis, overall HPV prevalence in sub-Saharan Africa was five times higher than in Europe, while HPV prevalence in South America and Asia was intermediate 6. Several population-based studies in China showed an obvious regional difference of HPV prevalence, and the overall HPV prevalence of Shanxi Province, Shenyang, Shenzhen and Beijing was 14.8%, 16.8%, 18.4% and 6.7%, respectively 7-10.

As a coal-rich province in China, Shanxi has a large number of female coal mine workers. Female coal mine workers usually have a relatively lower level of education, younger age of first sexual intercourse, multiple sexual partners, and poor hygiene habits, which have been demonstrated to be risk factors for HPV infection 11,12. With the humid and poor hygienic conditions in coal mine, it is favorable for micro-organism growth and propagation and harmful for the health of workers 11,13,14. Especially, female coal mine workers could be more prone to HR-HPV infection than the general population. However, epidemiological data on the prevalence and influencing factors of HR-HPV infection in female coal mine workers is scarce. Studies have been done for the association between HPV load and severity of cervical lesions with inconclusive results 15-18. Thus, in the current study, we aimed to explore the influencing factors of HR-HPV infection in female coal mine workers, and to evaluate the relationships between HR-HPV DNA load and cervical cytological and histological changes as well.

## Materials and Methods

### Study population and data collection

Between September 2011 and October 2012, 6,325 female coal mine workers (age between 20 to 65 years) were enrolled in our study. Subjects were considered eligible for the study according to the following criteria: a) Han people; b) married; c) age ≥20 years old; d) worked in coal mines for at least five years; e) resident in the coal mining area for at least two years; f) not pregnant or lactating; g) no prior history of cervical cancer or precancerous lesions; h) not suffering cancers during the enrollment and has no prior history of tumor of other systems; i) no prior history of treatments for cervix such as Loop Electrosurgical Excision Procedure (LEEP), conization and adnexectomy.

**Figure 1:**
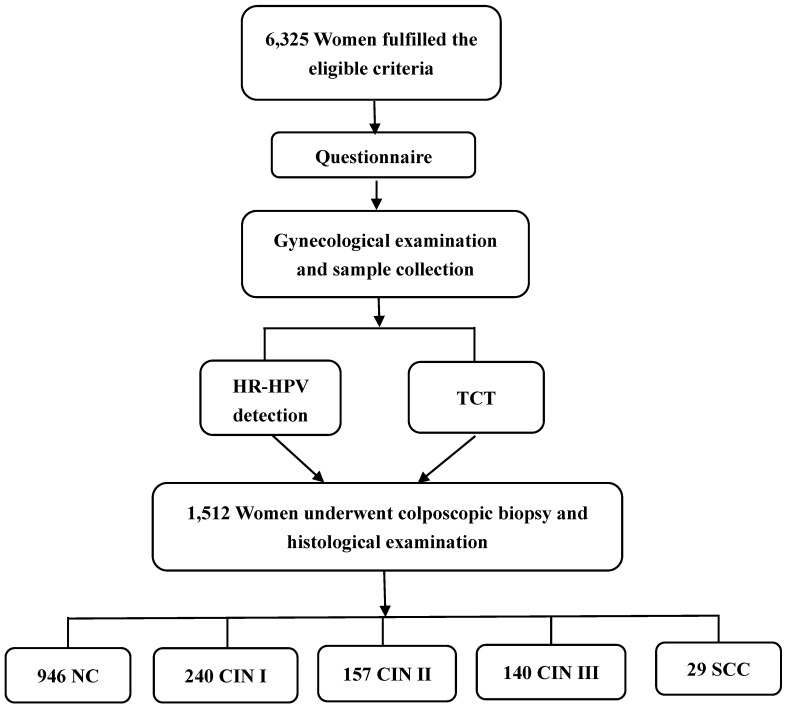
Flowchart of the study population.

All women enrolled in our study were interviewed by trained interviewers using the structured questionnaire regarding their socio-demographic information, working characteristics, reproductive information, history of gynecological diseases and family history of cancers. Then the subjects underwent a gynecological examination and had samples for HR-HPV detection and Thinprep cytology test (TCT) collected. Colposcopy with biopsy and histological examination were given to women with a positive diagnosis of HR-HPV and/or TCT (Figure 1).

Informed consents have been signed by all the participants, and this study has been approved by the Institutional Review Board of Shanxi Medical University (No. 2008LL07).

### HR-HPV Detection

DNA load of HR-HPV was evaluated by Digene second generation hybrid capture (HC2) DNA test, an in vitro acid hybridization assay with signal amplification using microplate chemiluminescence for HR-HPV DNA detection. A commercial kit detecting HPV types 16, 18, 31, 33, 35, 39, 45, 51, 52, 56, 58, 59 and 68 for performing HC2 was used. Alkaline solution was used to denature double-stranded DNA, and the liberated single-strand DNA combined with the RNA probe mix. RNA:DNA hybrids were then transferred to a capture microplate coated with goat polyclonal anti-RNA:DNA hybrid antibodies to be immobilized. Subsequently, alkaline phosphatase-conjugated antibodies to RNA:DNA hybrids were added to react with the bound RNA:DNA hybrids. After washing, chemiluminescent substrate, as the substrate for alkaline phosphatase, was added for signal amplification. Results were analyzed by DIGENE DML 2000 Software. DNA load was expressed by the unit of Relative Light Units/Cutoff Value (RLU/CO), representing the ratio of the light emission of a sample to the average of three positive control samples. HR-HPV DNA load of 1.0 (RLU/CO) or more was defined as HR-HPV positive. HR-HPV viral load was categorized into two groups: low and moderate viral load (<100 RLU/CO) and high viral load (≥100 RLU/CO) 16.

### TCT Test

Exfoliated cervical cells were collected for cytology test. The cytological evaluation was performed by two cytopathologists at Jincheng General Hospital and the Bethesda Classification system (2001 version) 19 was applied to evaluate cellularity. Diagnosis was classified as follows: a) negative for intraepithelial lesion or malignancy (NILM); b) atypical squamous cells of undetermined significance (ASC-US); c) low-grade squamous intraepithelial lesions (LSIL); d) high-grade squamous intraepithelial lesions (HSIL); e) squamous cell carcinoma (SCC). A diagnosis of ASC-US or worse was considered TCT test positive.

### Colposcopy and Cervical Histological Examination

Colposcopic biopsy was performed in subjects with a positive outcome of HR-HPV and/or TCT. Cervix was divided into quadrants and examined each quadrant by gynecological specialists. All visually abnormal areas were biopsied, and the quadrants without a visible lesion were given random biopsies at the squamo-columnar junction. Histological examinations by biopsy were evaluated together by two pathologists. The cases were classified as normal cervix (NC), cervical intraepithelial neoplasia grade 1 (CIN Ⅰ), grade 2 (CIN Ⅱ), grade 3 (CIN Ⅲ) and squamous cell carcinoma (SCC).

### Statistical Analysis

Influencing factors of HR-HPV infection were analyzed in women with different HR-HPV infection status (HR-HPV-positive and -negative), and associations between HR-HPV DNA load and cervical lesions were analyzed by comparing differences of HR-HPV DNA load in different cervical lesion groups. Count data were examined by Chi-square and trend Chi-square tests. One-way ANOVA was used to analyze differences between groups, and the differences between any two groups were further compared by SNK test. Multivariate unconditional logistic regression was used to determine the influencing factors of HR-HPV infection, and backward stepwise likelihood was used with the inclusion criteria of 0.05 and the exclusion criteria of 0.10. All the data analyses were performed using Statistical Package for the Social Sciences (SPSS) software version 19.0 (IBM Corporation, Armonk, New York, USA). All reported *p* values were two-sided, and statistical significance was defined as *p*<0.05.

## Results

### Socio-demographic characteristics related to HR-HPV infection

The mean age of our study subjects was 35.31±8.89 years (ranged between 20 and 65 years). Subjects aged between 30 and 50 years old had the highest HPV prevalence (26.5%), and the prevalence declined as age increased. Only 1,850 women (29.2%) received higher education (college/university and above). The HR-HPV infection rate significantly decreased with increasing education level (*χ*^2^_trend_=35.897, *p*<0.001). More than half of the women (3,572/6,325, 56.5%) reported born in Jincheng and the majority (5,230/6,325, 82.7%) were married. However, HR-HPV infection rate showed no significant association with birthplace and marital status (*p*>0.05). The socio-demographic characteristics of the study subjects are shown in Table 1.

### Influencing factors related to HR-HPV infection

Of the 6,325 participants, 1,405 (22.2%) were HR-HPV positive. Univariate analysis showed a significant association between HR-HPV infection and workplace, menopause, contraception, contraceptive method, gravidity, parity, abortion history, artificial abortion history, and the history of gynecological disease (vaginitis, pelvic inflammation disease, uterine fibroids, hyperplasia endometrii, and ovarian tumors) (*p*<0.05) (Table 2). Multivariate unconditional logistic regression showed that contraception, artificial abortion, working inside the mines and history of gynecological diseases were risk factors for HR-HPV infection, while menopause was a protective factor (Table 3).

### HR-HPV DNA load according to cervical cytological and histological changes

**Figure 2:**
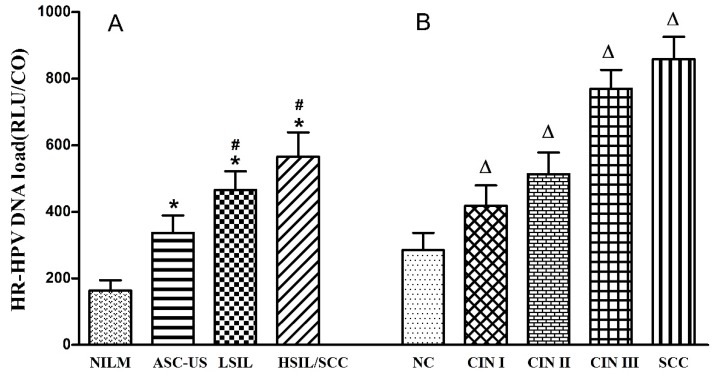
HR-HPV DNA load among different cytological (A) and histological (B) changes. HR-HPV load increased with severity of cervical cytological and histological changes. *, Difference showed a statistical significance when compared to NILM (*p*<0.05); #, difference showed a statistical significance when compared to ASC-US (*p*<0.05); Δ, difference showed a statistical significance when compared to NC (*p*<0.05).

In all 6,325 subjects, colposcopy with biopsy and histological examination were given to 1,512 women with a positive diagnosis of HR-HPV and/or TCT. Among them, 946 were diagnosed with NC, 240 with CIN Ⅰ, 157 with CIN Ⅱ, 140 with CIN Ⅲ and 29 with SCC. Associations between HR-HPV DNA load and cytological and histological changes are shown in Table 4 and Figure 2. HR-HPV DNA load increased with the severity of cervical cytological and histological changes (*p*<0.05). High viral load of HR-HPV was found to be significantly related to cytological (*χ*^2^_trend_=177.372, *p*<0.001) and histological (*χ*^2^_trend_=194.501, *p*<0.001) changes of cervix.

## Discussion

In this study, we found that female coal mine workers had a relatively higher prevalence of HR-HPV with 22.2%, which was higher than population-based prevalence observed in Shanxi Province (12.2%), Shenyang (11.7%), Shenzhen (13.5%) and Beijing (5.8%), suggesting that female coal mine workers might have a higher risk for cervical cancer 7-10.

In our study, HR-HPV prevalence in female coal mine workers reached peak at ages 30-50, which might be attributable to the relatively active sex life in women of this age as several studies have shown that HPV could be transmitted to cervix efficiently by vaginal intercourse 20. We also found that HR-HPV infection rate decreased with increasing education level, which is in consistent with previous studies 21. Shift work could result in a series of negative effects on health, such as adverse effects on hormone and reproductive function of females 22. In this study, the HR-HPV infection rate in shift workers was higher than non-shift workers, though it didn't reach a statistical level. Improving the working environment of the coal mines and developing cervical cancer screening strategies for coal miners may be particularly efficient. In addition, women working inside the mines have 1.230 higher risk of HR-HPV infection than workers working outside the mines, which may be due to the fact that the humid and poor hygienic conditions in the mines can promote the growth of microbe 11,13,14.

In our study, significant increase in the risk of HPV infection was observed in women using contraceptives, who have 1.395 higher risk of HR-HPV infection than women who did not, which is similar to a former study 23. Majority (86.5%) of the subjects in our study chose intrauterine device (IUD) and oral contraceptives (OC) as contraceptive methods. Abnormal bleeding induced by IUD could increase the risk for HPV infection. Moreover, OC has been shown to enhance hydroxylation of estradiol to 16α-hydroxyestrone in cervical cells infected with HR-HPV, which induces increased transcription of the HPV oncogenes E6 and E7 24. In addition, menopause was found to be a protective factor of HR-HPV infection in our study, and the HR-HPV risk in postmenopausal women was only half of that in premenopausal women. Our previous studies have shown that menopause was a protective factor, which could be ascribed to the decreased estrogen level of menopause women 25,26. Therefore, we suggest the use of condoms instead of OC or IUD for contraception to effectively reduce HPV infection.

Our results showed that artificial abortion was also a risk factor for HR-HPV infection. Artificial abortion can cause damage to the endometrium and mechanical injury to the cervix, so as result in HR-HPV infection. In this study, history of gynecological diseases was also a risk factor for HR-HPV infection. Several studies reported that women with gynecological diseases were prone to persistent HR-HPV infection due to unstable sexual hormone levels, poor function of immune system, lower resistance to HR-HPV and weak ability of viral clearance 27,28. Our results suggested that active treatment for gynecological diseases is of great importance to reduce the risk for HR-HPV infection.

Results of this study also showed that HR-HPV DNA load and the proportion of high viral load increased with the severity of cervical cytological and histological changes. Huang et al 29 reported that the risk of cervical precancerous lesions and cervical cancer significantly increased when HPV DNA load was higher than 100 RLU/CO and 500 RLU/CO respectively, which was consistent with our results. Dalstein V et al 30 reported that high viral load could be used as a short-term marker of progression towards CIN from a prospective study. Moreover, another cohort study revealed that the progression to CIN Ⅱ/Ⅲ was linked to HPV 16 DNA load which evaluated by quantitative real-time PCR, and the dynamic change of HPV viral load was associated with cervical lesions 31. Peitsaro et al 32 reported that high viral load was likely to increase the risk for viral integration with the host, and this could partly explain the relationship between viral load and cervical lesions. Furthermore, the possible use of the HPV load to predict the degree of cervical lesion has been proposed 33,34. It is beneficial to carry out antivirus therapy in women with high HR-HPV load to reduce the risk for cervical cancer in this population.

In conclusion, in this study we assessed the influencing factors of HR-HPV infection in female coal mine workers, and found that contraception, artificial abortion, working inside the mines and gynecological diseases could increase the risk of HR-HPV infection. HR-HPV DNA load could predict the risk of cervical precancerous lesions and cancer based on this cross-sectional study. Our findings may provide scientific basis to reduce the risk of HR-HPV infection and cervical cancer in this vulnerable population. Certainly, out of the limitations of the research method we used, the ability to prove the hypothesis of etiology was relatively weak. The prospective cohort studies will be needed to provide more powerful evidence.

## Figures and Tables

**Table 1:** Socio-demographic characteristics related to HR-HPV infection by univariate analysis.

Variable	n	HR-HPV infection rate (%)	*χ*^2^	*p*	OR (95%CI)
**Age(years)**					
20-	857	15.1			1.000
30-	2156	26.3	43.662	<0.001	2.014(1.632-2.485)
40-	2134	26.7	46.406	<0.001	2.057(1.667-2.538)
50-	773	12.4	2.060	0.515	0.812(0.611-1.079)
≥60	405	10.6	4.596	0.032	0.670(0.464-0.968)
**Education level**					
Junior high school and below	1939	25.9			1.000
Senior high school*	2536	22.7	6.064	0.014	0.841(0.733-0.965)
College	768	18	19.119	<0.001	0.627(0.508-0.774)
University and above	1082	17.5	27.922	<0.001	0.606(0.503-0.730)
**Birthplace**					
Jincheng	3572	22.3			
Other cities	2753	22.1	0.047	0.829	1.013(0.899-1.142)
**Marital status**					
Married	5230	22.6			
Divorced/separated/widowed	1095	20.4	2.618	0.106	1.142(0.972-1.341)

n, number; OR, odds ratio; CI, confidence interval; *, Technical secondary school degree is included in Education level of senior high school.

**Table 2:** Potential influencing factors related to HR-HPV infection by univariate analysis.

Variable	n	HR-HPV infection rate (%)	*χ*^2^	*p*	OR (95%CI)
**Workplace**					
Outside the mines	3255	18.8			1.000
In the mines	3070	25.9	45.966	<0.001	1.509(1.339-1.700)
**Shift work**					
No	1587	20.9			1.000
Yes	4738	22.5	1.721	0.19	1.097(0.955-1.261)
**Menopause**					
No	5434	21.9			1.000
Yes	891	15.3	20.341	<0.001	0.642(0.529-0.780)
**Contraception**					
No	1860	19.6			1.000
Yes	4465	23.3	10.227	<0.001	1.244(1.088-1.422)
**Contraceptive method**					
Condom	601	13.5			1.000
IUD	2284	51.2	275.472	<0.001	5.731(5.252-8.626)
OC	1580	35.4	100.735	<0.001	3.515(2.721-4.540)
**Gravidity**					
≤1	2577	20.3			1.000
2-3	3349	21.9	2.210	0.137	1.100(0.970-1.248)
>3	399	37.1	61.362	<0.001	2.422(1.932-3.037)
**Parity**					
≤1	5520	21.7			1.000
≥2	805	25.8	6.950	0.008	1.257(1.060-1.490)
**Abortion history**					
No	2993	19.2			1.000
Yes	3332	24.9	29.631	<0.001	1.395(1.237-1.573)
**Artificial abortion**					
No	3371	23.1			1.000
Yes	2954	25.5	37.272	<0.001	1.439(1.28.-1.618)
**History of gynecological diseases**					
No	5897	21.6			1.000
Yes	428	30.4	17.770	<0.001	1.583(1.277-1.963)

n, number; OR, odds ratio; CI, confidence interval; IUD, intrauterine device; OC, oral contraceptives.

**Table 3:** Influencing factors for HR-HPV infection assessed by multivariate unconditional logistic regression.

Variables	*β*	S_b_	Wald *χ*^2^	OR^*^ (95% CI)
Menopause	-0.629	0.112	73.102	0.402(0.306-0.507)
Contraception	0.196	0.079	4.961	1.395(1.102-1.458)
Artificial abortion	0.512	0.152	9.362	1.603(1.202-1.856)
Working inside the mines	0.183	0.105	6.587	1.230(1.056-1.528)
History of gynecological diseases	0.175	0.120	2.018	1.198(1.001-1.462)

OR, odds ratio; CI, confidence interval; ^*^, adjusted OR for age and education level.

**Table 4:** Relationships between HR-HPV DNA load and cervical lesions

Cervical lesions	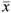 ±s	HR-HPV DNA load		*χ^2^*	*p*	OR (95%CI)
		L (%)	H (%)			
***Cytological change***						
NILM	163.850±30.170	4505(77.5%)	1308(22.5%)			1.000
ASC-US	336.780±51.961*	169(58.0%)	122(42.0%)	58.280	<0.001	2.486(1.954-3.163)
LSIL	465.170±56.192*#	63(45.4%)	76(54.6%)	78.750	<0.001	4.155(2.959-5.835)
HSIL/SCC	565.230±73.557*#	33(40.4%)	49(59.6%)	63.331	<0.001	5.114(3.275-7.986)
	F=44.848, *p*<0.001	*χ*^2^=183.394*, p<*0.001;* χ*^2^_trend_*=*177.372*, p*<0.001				
***Histological change***						
NC	284.60±52.108	618(65.3%)	328(34.7%)			1.000
CIN Ⅰ	417.88±61.102Δ	109(45.4%)	131(54.6%)	31.991	<0.001	2.264(1.699-3.018)
CIN Ⅱ	513.12±64.587Δ	57(36.3%)	100(63.7%)	47.761	<0.001	3.306(2.325-4.699)
CIN Ⅲ	769.51±56.680Δ	16(11.4%)	124(88.6%)	145.809	<0.001	14.809(8.530-24.997)
SCC	859.32±65.596Δ	3(10.3%)	26(89.7%)	36.784	<0.001	22.481(4.906-54.354)
	F=12.440, *p*<0.001	*χ*^2^=199.176, *p*<0.001; *χ*^2^_trend_=194.501, *p*<0.001				

OR, odds ratio; CI, confidence interval; *, Difference showed a statistical significance when compared to NILM (*p*<0.05); #, difference showed a statistical significance when compared to ASC-US; Δ, difference showed a statistical significance when compared to NC; L, low and moderate HR-HPV DNA load; H, high HR-HPV DNA load.
